# Cross-recurrence quantification analysis of categorical and continuous time series: an R package

**DOI:** 10.3389/fpsyg.2014.00510

**Published:** 2014-06-27

**Authors:** Moreno I. Coco, Rick Dale

**Affiliations:** ^1^Faculdade de Psicologia, Universidade de LisboaLisboa, Portugal; ^2^Cognitive and Information Sciences, University of CaliforniaMerced, CA, USA

**Keywords:** cross-recurrence analysis, cognitive dynamics, methodology comparison, behavioral data, R library

## Abstract

This paper describes the R package crqa to perform cross-recurrence quantification analysis of two time series of either a categorical or continuous nature. Streams of behavioral information, from eye movements to linguistic elements, unfold over time. When two people interact, such as in conversation, they often adapt to each other, leading these behavioral levels to exhibit *recurrent states*. In dialog, for example, interlocutors adapt to each other by exchanging interactive cues: smiles, nods, gestures, choice of words, and so on. In order for us to capture closely the goings-on of dynamic interaction, and uncover the extent of coupling between two individuals, we need to quantify how much *recurrence* is taking place at these levels. Methods available in crqa would allow researchers in cognitive science to pose such questions as how much are two people recurrent at some level of analysis, what is the characteristic lag time for one person to maximally match another, or whether one person is leading another. First, we set the theoretical ground to understand the difference between “correlation” and “co-visitation” when comparing two time series, using an aggregative or cross-recurrence approach. Then, we describe more formally the principles of cross-recurrence, and show with the current package how to carry out analyses applying them. We end the paper by comparing computational efficiency, and results’ consistency, of crqa R package, with the benchmark MATLAB toolbox crptoolbox (Marwan, 2013). We show perfect comparability between the two libraries on both levels.

## 1. Introduction

We describe an analytic framework for studying how human behavior is organized in time, with an emphasis on linguistic interaction. Interaction, and perhaps all human behaviors, are organized in systematic and interesting ways in time, and it is important to explore new techniques to help researchers examine this temporal organization.We introduce cross recurrence quantification analysis (CRQA), a technique growing in use in many fields. This analysis framework may contribute to areas of cognitive science which have not always looked closely to behavioral organization *in time*. Many studies utilize atemporal methods, which *aggregate* over temporal dimensions of analysis, often focusing instead on the magnitudes of behaviors that encompass interaction. For this discussion, we refer to “aggregative” as any analysis that averages behavior across time, thereby abstracting over the temporal ordering of interactive behaviors, and focusing instead on the rate, or magnitude, of occurrence.

This aggregative approach has borne considerable fruit for some questions. For example, when two people interact they may come to mimic each other as measured by behavioral frequencies (Bargh and Chartrand, 1999), and they may utilize similar sentence structures at opportune times as discerned by careful experimental design (Haywood et al., 2005). Many papers have shown that humans can coordinate syntactic structures (Branigan, 2007), entrain on descriptions (Brennan and Clark, 1996), spatial perspective (Schober, 1993), and so on. Indeed, this aggregative approach has been the dominant technique in the language sciences for studying the convergence of human interlocutors (we discuss prominent exceptions later in this paper).

There is no doubt that such aggregative methods are important, and often sufficient for rendering new insights into interaction. But recent work has sought to characterize the manner in which these aggregate scores unfold. Put simply, taking aggregate measures and “unfolding them in time” offers both intriguing methods, and also new questions: Does the temporal organization of interaction show interesting patterns, beneath their aggregation? Do these patterns shed light on the mechanisms underlying human interaction? How are different behavioral measures organized in time relative to each other? What variables impact the shape of coordination between two people who are interacting?

By unfolding behavioral measures, and subjecting them to temporal analysis, we can indeed find distinct dynamics between two interacting people. For example, Richardson and Dale (2005) find that when one person is speaking to a listener, they exhibit coupled gaze patterns, but with the listener’s eye movements lagged by a characteristic time of about 2 s. Interestingly, the lag time of any one listener predicted their comprehension; the dynamics of coupling revealed comprehension. But as two people talk bidirectionally (taking turns as speaker and listener), this lag time approaches 0 s, suggesting tighter coupling occurs during real-time interaction (Richardson et al., 2007; Dale et al., 2011a). And beyond just eye movements, other behavioral aspects of interaction exhibit this coupling, such as nods, gestures, and conversational moves (Louwerse et al., 2012).

These basic insights were generated through what is called *cross-recurrence* methods. It is a family of techniques measuring how and the extent to which streams of information come to exhibit similar patterns *in time*. This analysis framework was developed, and is extensively employed, in the natural sciences in such diverse domains as heart rate variability, seismology, and chemical fluctuations (see Marwan et al., 2007; Marwan, 2008, for reviews). In psychology, it rapidly gained attention in the domain of motor control (e.g., Richardson et al., 2005; Shockley and Turvey, 2005; Stephen et al., 2009), being applied to both within- and between-person dynamics, such as during precision-target tasks (Balasubramaniam et al., 2000) and even conversation (Shockley et al., 2003).

As we describe further below, the method is often referred to as a “non-linear” technique that permits the researcher to avoid certain assumptions that linear statistics make (see Riley and Van Orden, 2005). This method can also reveal system characteristics, phrased in the language of dynamical systems, permitting researchers to describe their phenomena in new and potentially interesting ways. A comprehensive review of the method can be found in Marwan et al. (2007), an especially lucid introduction to it in Webber and Zbilut (2005), and a description of the method’s broader context in dynamical systems and psychology in Richardson et al. (2014). An excellent MATLAB toolbox for recurrence can be found in Marwan (2013)^1^.

In this paper, we present crqa, a package written in R implementing basic methods to perform cross-recurrence analysis. Even if the crqa package can be technically used with any stream of temporal data, we designed the crqa package mainly to investigate human behavioral dynamics, such as eye-movement patterns or conversational moves, emerging during linguistic interaction. For this reason, we explain the theoretical principles of cross-recurrence analysis, as well as demonstrate the package’s functionalities, emphasizing the value of this technique for studying linguistic interaction: finding temporal patterning between two persons as they interact.

We start in an unusual but, we believe, helpful manner: by motivating the importance of unfolding aggregate measures, and showing how recurrence does this. To do so, we make use of highly simplified simulated models as demonstration (cf. Beer, 2003), where hypothetical data are generated from known principles. Then, we provide more formal details about CRQA and the way it is computed, then explain the most important functions implemented in the crqa library and briefly describe the data available to test it. Finally, we compare the computational accuracy and efficiency of our R package with the state of the art MATLAB toolbox, crptoolbox (version 5.15) by Marwan et al. (2007) on simulated dichotomous time series. We report tests of the computational *efficiency* (user elapsed time) of the libraries as a function of the length of the time series and *consistency* (absolute difference and correlations) of the measures obtained by the two libraries.

## 2. Motivating recurrence: aggregation, covariance, and co-visitation

In this section, we aim to briefly motivate cross-recurrence methods, and relate them conceptually to statistical aggregation (“atemporal” aggregation), and cross-correlation approaches. We will not articulate the formal relationships among these analyses, as they have been articulated elsewhere (see Marwan et al., 2007; Bakeman and Quera, 2011; Dale et al., 2011b). However, there are relatively few clear comparisons of these techniques that explain where and when each would be useful. Aggregation and correlation scores are highly useful and easy to compute, but they are not a comprehensive characterization of two systems’ relative behaviors. By focusing on the path of a system’s behavior in time, there may be other indices that describe how two systems are exhibiting similar or dissimilar patterns. We hope this simple section motivates the distinction between covariance-based and “visitation-based” measures.

We use a simple toy model which derives from a common experimental circumstance. Imagine having a confederate (C) interact with 40 subjects (S) in the laboratory. In one condition (high), you have the confederate amplify a particular pattern of behavior, such as scratching the face or touching the foot. In another condition (low) you have them minimize such behaviors. Doing an experiment much like this, Bargh and Chartrand (1999) had confederates use non-salient and seemingly incidental behaviors to induce this behavior in a communication partner. By having a confederate engage in one or the other behavior, they can induce the participant to increase their behavior along the same dimension. Researchers aggregate the observed effect on participants (how many times the participant engages in these behaviors), and find that the rate can be amplified as a function of the confederate’s behavior (high vs. low rate of target behavior).

**Figure 1:**
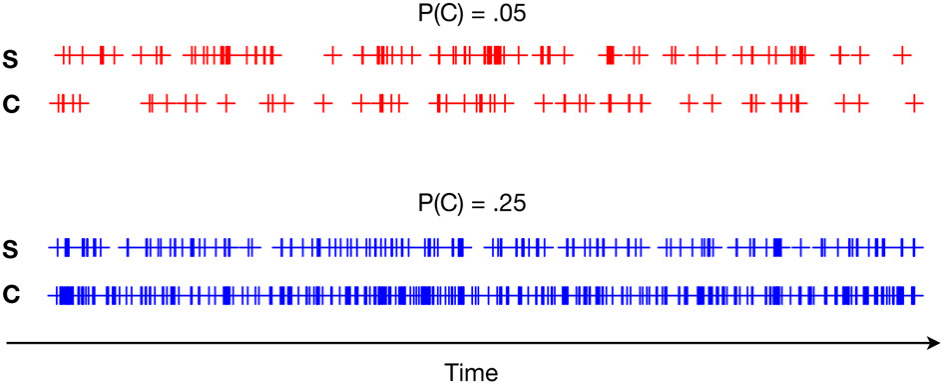
**Two example experimental runs, in which we observe the behavior of two simple conversational “agents,” a confederate (C) and participant (S), over 1000 time steps**. The confederate’s behavior is experimentally setup to amplify the occurrence of the event. P(C) in the plot reflects the raw probability that the confederate will emit the behavioral event (see Table 1). As specified in the agent’s policies, an increase in the behavioral event by the confederate should also increase it in the participant, which analyses are meant to bear out.

Let us take up some purely hypothetical data for the sake of demonstration, using precisely this setup. We designed a very simple simulation of the kind just described, in which we simulate data about the occurrence of a specific behavioral event, across time, between confederate and participant “agents.” We use simts code, available in the crqa package, to specify the behavior of confederate vs. participant along some dimension in Table 1. In actual practice, these data may be the occurrence of touching the face or foot (Bargh and Chartrand, 1999), looks to certain characters on a computer screen (Richardson and Dale, 2005), or an entire array of behaviors from dialog moves to laughter events (Louwerse et al., 2012). Readers may consult detailed advice and coding schemes for discrete behaviors in Bakeman (1997). Here we will simply call this an “event” and track its occurrence over time, for two agents, as shown in Figure 1.

**Table 1:** **A simple algorithm for producing a system (C) that drives a second system (S) for a binary time series (1 for event occurrence; 0 otherwise)**.

**Variables**	**Algorithm**
P(X) = base rate of event for person X	Produce a time series for C and S events:
P(X|Y) = rate of event for X given Y did	
P(X|X) = probability of event repetition	Do 1000 times If rand < P(C) C outputs event (=1) Else if rand < P(C|C) and C = 1 C outputs event Otherwise C outputs no event (=0) If rand < P(S|C) and C = 1 S outputs event (=1) Else if rand < P(S) S outputs event Else if rand < P(S|S) and S = 1 S outputs event Otherwise S outputs no event (=0)

*The algorithm is available in the package* crqa, *as function* simts. *A practical explanation about implementation and usage of the function* simts *can be found in the Supplementary Material of this paper*.

*Notes: In the algorithm, C = confederate agent, S = participant agent. 20 such runs were conducted for 1000 iterations for each of conditions low P(C) = 0.05 and high P(C) = 0.25. Other parameters include: P(S) = 0.05, P(C|C) = P(S|S) = 0.2, and P(S|C) = 0.25. Parameters were chosen to bring average behavior to Supplementary Material in the low condition. This is merely for demonstration and other parameter values would work fine*.

**Figure 2:**
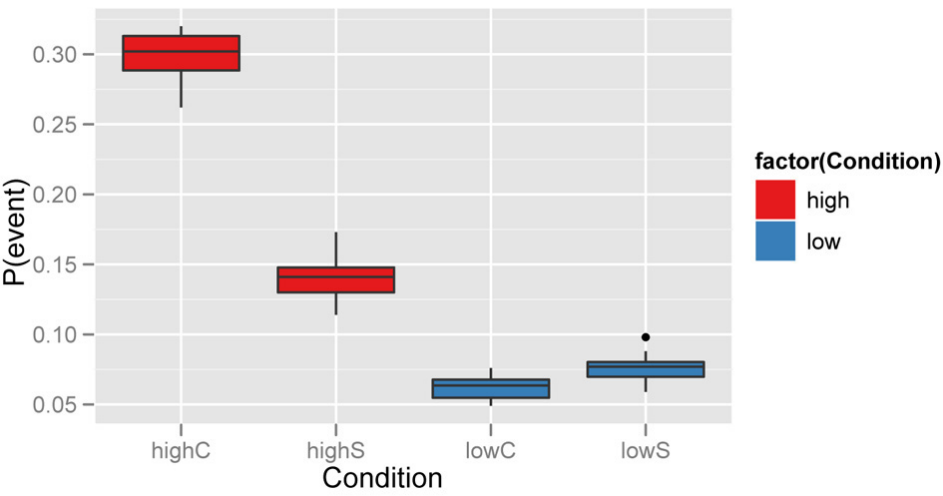
**Data from 20 simulated interactions for each condition of the confederate’s event occurrence rate (0.05 vs. 0.25)**. As expected, one sees a relative increase in the event’s occurrence in agent S if it occurs in agent C.

The raw data that this study would use, presumably, is a proportion, aggregated over time, of the behavioral event of interest. In Figure 2, one can see that these events are then aggregated into one rate score. The left side of the plot shows a relatively higher incidence of the behavioral event by the participant agents, compared to the right side of the plot. In our simplified conversational agents, this is a result of the fact that the confederate agents can drive the probability of the event of interest in the participant agent.

**Figure 3:**
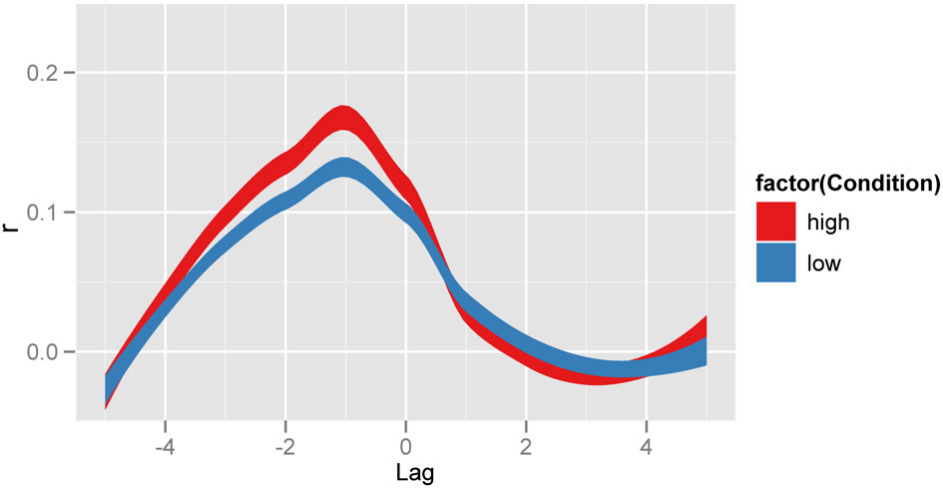
**Unfolding aggregate scores using cross correlation**. Cross-correlation functions between confederate and participant agents. The high agent condition (red), reflecting the cross-correlation between C and S agents at different time lags or shifts (scale: step increments), shows maximal variance accounted for at lag −1, C leading S by one time step (as set in the simulation). Smoothed profiles generated with ggplot2 in R, with stat_smooth which uses standard error to define the width of the lines.

Another way of achieving this distinction between low and high conditions is to observe the *correlation* between their behavior and that of the confederate. This is shown in Figure 3, which displays the Pearson correlation between interlocutors at different time lags. Such a cross-correlation function gives a more detailed picture of the temporal interaction between interlocutors. The maximal correlation (≈0.2) occurs at a lag of −1, which reflects the confederate agent leading the participant agent^2^. Because a higher event occurrence P(C) generates more events in agent S, the variance accounted for at that lag will also significantly increase, as more events in the confederate will be the driver of those in the participant. Recent exciting extensions of this technique can use a windowed approach to visualize and explore temporal relations, as shown by Boker et al. (2002) and Barbosa et al. (2012). In general, cross-correlation informs about the relative covariation between event sequences (i.e., coupling), and its maximal point (in our example, C leads maximally S at lag −1).

**Figure 4:**
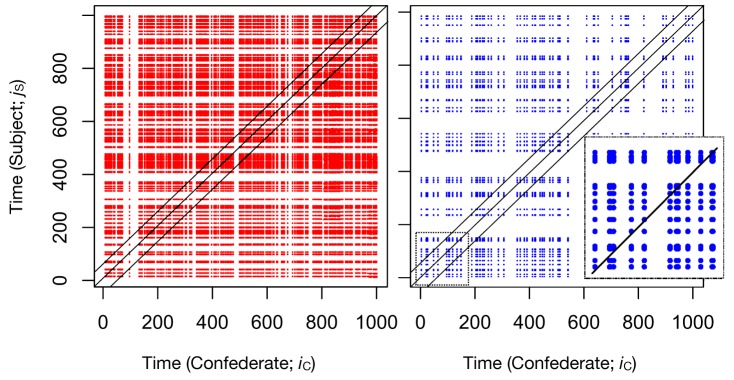
**Example cross recurrence plots (CRPs) of two sample runs of the simulated data**. Left shows a high condition run, right shows a low condition run. Points reflect relative moments in time where C and S are revisiting event states (=1), whereas 0’s (non-events) do not produce points on the plot. Three black lines define the approximate location of the lag calculations described in the text (from −5 to +5). The middle black line is the line of coincidence (LOC), where lag = 0. Though difficult to see in this plot, the points appear shifted slightly upwards (lagged +1), indicative of C leading S. This pattern becomes more evident in Figure 5, when calculating percentage recurrence over these diagonals.

This correlation measure has some similarities to aggregation, and be described as “co-aggregation,” i.e., observing how the rate of a behavior co-varies with that of another time series. Covariation methods are obviously useful and fruitfully applied in many contexts, but even beyond correlation there are many temporal patterns worthy of exploring. In the cross-recurrence case, one may be said to be exploring co-visitation patterns: How one time series is *revisiting* states that the other time series has visited. This works by quantifying the pattern of visitation of the two systems, rather than simply quantifying their relative rate of occurrence. First, imagine plotting all points (*i*_*C*_, *j*_*S*_) where *i*_*C*_ are the *time indices* of the event in agent C’s time series, and *j*_*S*_ are the indices of the event in agent S. This produces a visualization of the pattern of co-visitation over time between the two systems. This is shown in Figure 4. These are referred to as cross-recurrence plots (CRPs).

Cross-recurrence quantification analysis (CRQA) is the quantification of the patterns of co-visitation taking place on these plots. Already, one can simply see that there is a much greater density of points on the high condition plot than the low condition. Here we show that quantification of the plots can obtain similar information to cross-correlation, but under a different interpretive scheme. In fact, as we show in the next section, there is a whole range of measures that can be extracted from these plots, and they can become quite sophisticated in their potential implications for the properties of cross recurrence taking place between the two systems that are being compared.

**Figure 5:**
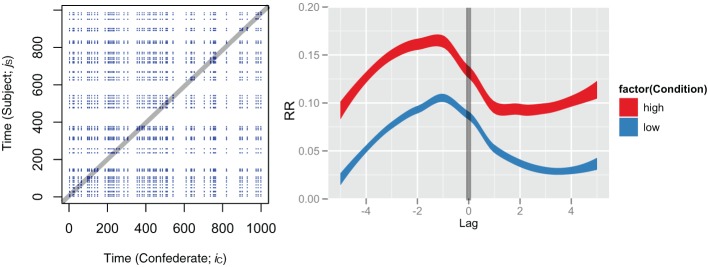
**By calculating the rate of points on diagonals around the LOC (left side), we obtain a *diagonal-wise recurrence* that reflects the relative co-visitation, as a function of lag (right side)**. The line superimposed on both panels shows the approximate region over which percentages are calculated. Like cross-correlation we get a maximization at −1, reflecting C driving S. However, the difference between the conditions is larger, proportional to the relative rate of occurrence. Recurrence does not count non-events (0’s), so the *y*-axis levels will be determined by the frequency of the events in the time series (1’s). Smoothed profiles (on the right) were generated with ggplot2 in R, with stat_smooth which uses standard error to define the width of the lines.

The line of coincidence (LOC) on this plot is where *i*_*C*_ = *j*_*S*_, where the points reflect the systems doing the same thing at the same time. By calculating the *rate* of the event recurrence along the diagonals around the LOC, we obtain a diagonal-wise recurrence rate (RR) measure that also provides a functional characterization of coupling (again, maximized at −1). However, the results will be more directly influenced by the rate of co-visitation, or recurrence. So, while cross-correlation gives a general measure of the co-variation between two series, cross recurrence shows a co-visitation score that will vary across experimental conditions. This is evident in the diagonal-wise RR profile shown in Figure 5, right panel.

Though this simple diagonal-wise RR profile correlates with cross-correlation (especially in these simple cases), the overall measures will behave differently depending on the rate of occurrence of events in the time series. It is also important to note that cross recurrence provides the researcher an option to remove the non-event matches (0’s), whereas in cross-correlation they are preserved and explicitly counted toward co-variation (for discussion see Dale et al., 2011b)^3^.

Finally, and importantly, measures so far are *descriptive* in nature, in the statistical sense that they are not inferential. In order to draw inferences regarding the differences between conditions, there are a variety of techniques that are relevant. In a research context, one collects dozens of dyads or individual subjects from whom time series are drawn. Comparing average recurrence profiles can be done by reference to certain baselines. Richardson and Dale (2005) use both surrogate (“virtual pairs”) and shuffling techniques to compare the observed profiles against these null cases. Shockley et al. (2007) also use the surrogate approach, but on continuous body-motion data. Another approach is simply to compare aggregate measures between two or more experimental conditions (e.g., Shockley et al., 2003). In general, with categorical time series of the kind we show here, shuffling the time series produces approximately the same expected mean as surrogate pairing (Dale et al., 2011a). In continuous time series (discussed further below) shuffling should never be the basis of a baseline, and a random pairing of virtual pairs is the preferred approach. Recently, growth-curve analysis may afford a way of modeling these profiles that would avoid problems of the baseline. For example, by testing the significance of various coefficients in a polynomial time model, one can determine whether a significant quadratic trend is present. We would endorse this as an important next step in establishing an inferential basis for these profiles, and the reader can consult (Mirman, 2014)^4^.

Below we go beyond this simple diagonal-wise RR measure, showing that CRQA also affords an array of other measures to characterize coupling between time series. And in fact, most of these other measures have no obvious analog with the cross-correlation function. These properties have led some to refer to CRQA as a “generalisation of the linear cross-correlation function” (Marwan et al., 2007, p. 256).

Here we have used a simple toy system to compare and contrast aggregation, co-variation, and co-visitation analyses. If one is simply interested in raw rates of occurrence, then aggregation is adequate. However, if the researcher wishes to explore functional relationships between systems, cross-correlation or cross-recurrence methods may shed detailed temporal light on their relationship. Cross-correlation measures aggregate co-variation between the two systems, and the maximal correlation observed reflects a stable coupling function between the two systems. However, it does not preserve relative rate of “co-visitation” of event states by the two systems. A similar source of information about coupling can be obtained by calculating diagonal-wise RR from cross-recurrence plots, providing both a coupling function *and* a relative rate of occurrence of one system visiting the events of another. As just noted, this is just one simple measure among many provided by CRQA.

Now that we have motivated the basic interpretive frameworks afforded by these analyses, we delve into CRQA in the next sections and detail how to use the R library.

## 3. Principles of CRQA

As sketched in the last section, cross-recurrence quantification analysis has been developed to capture the recurring properties and patterns of a dynamical system, which results from two streams of information interacting over time (Zbilut et al., 1998). In behavioral sciences, such streams of information can either be as “concrete” as body sways or eye-movement trajectories, and even heart rate (Shockley et al., 2003; Richardson and Dale, 2005; Wallot et al., 2013), but they can also be more “abstract” sequences of linguistic information, such as the words exchanged by two interlocutors during a dialog (for a recent review see Fusaroli et al., in press).

CRQA may thus shed light on the information-feedback dynamics occurring while actions (non-linguistic, linguistic) are transmitted, received, and responded to incrementally by participants in dialog. So, in the context of a communicative task, CRQA quantifies, for example, how much delay is needed for a listener to be maximally aligned to the instruction delivered by the speaker, how much alignment is observed overall, and so on.

**Figure 6:**
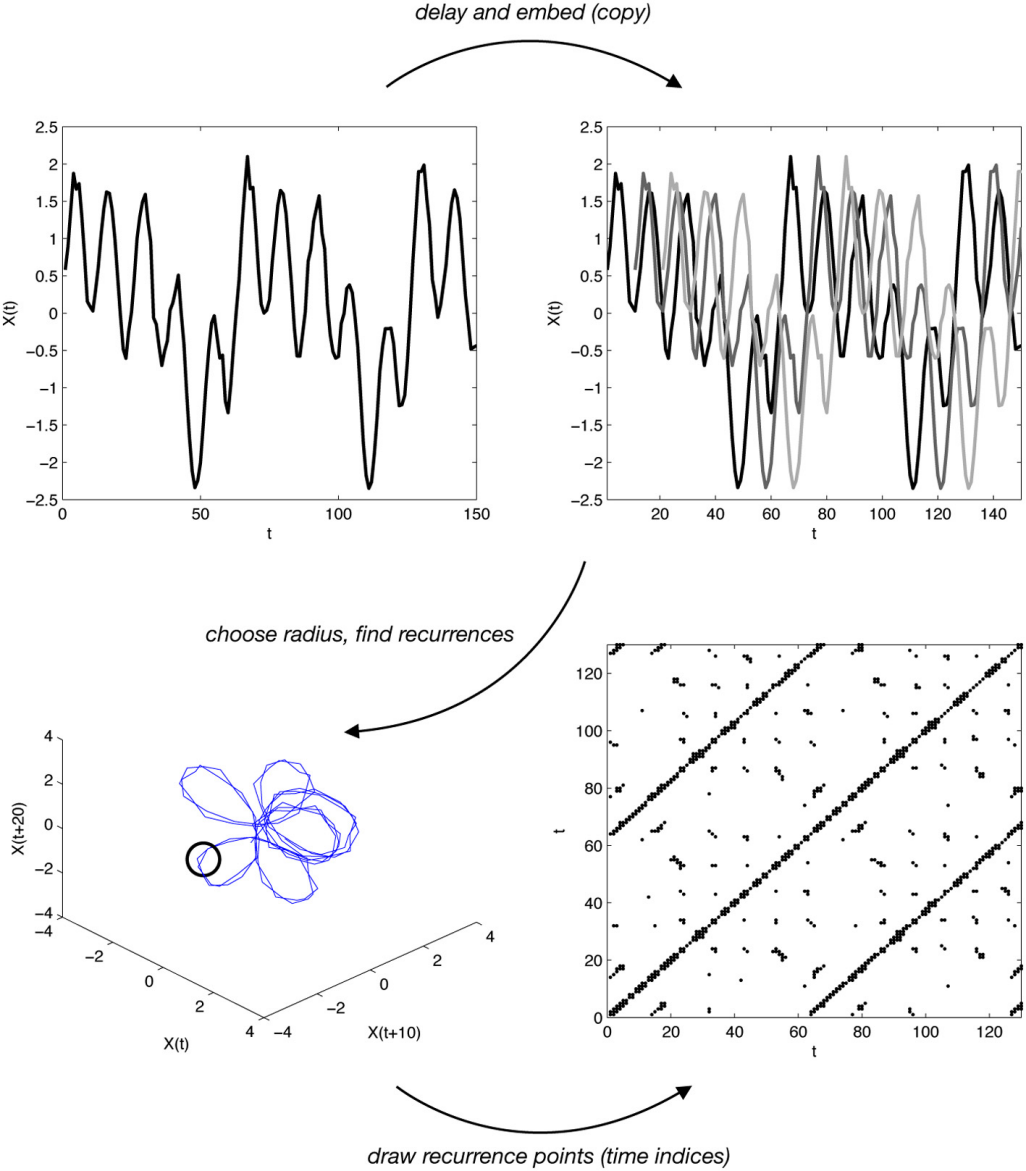
**A basic sketch of how recurrence is constructed from one time series (top left)**. The time series is lagged (by 10), copied (3 times), and overlaid with itself **(top right)**. If we use 3 dimensions (copies), then it is possible to visualize this reconstructed phase space **(bottom left)**. By drawing a radius of a given size around parts of this reconstructed phase space (thick line, **bottom left**), one can determine when recurrence is taking place. The time indices of these recurrence points can be used to construct the recurrence plot **(bottom right)**. Cross recurrence is done in almost exactly the same way, except two time series are used.

Usually CRQA is explained by reference to concepts from dynamical systems. We assume to have measured a time series—one measurement sampled over time—from two systems. Though this single measurement is probably a one-dimensional scalar, CRQA starts by overlaying delayed copies of this time series, for each system separately (displayed in the top row of Figure 6, illustrating this process for one time series). CRQA compares two time series by calculating the degree of their recurrence when these delays are introduced with different numbers of copies, or “embedding dimensions.” Specifically, from an original time series *X*(*t*), delayed copies *X*(*t* + τ) are generated by introducing a lag τ into the original time series. The different dimensions of embedding are obtained by considering multiple lags *X*(*t* + *m*τ).

If in 2 or 3 dimensions, we can plot this delay/copy process, as shown in the bottom-left of Figure 6. This is often referred to as a system’s “reconstructed phase space.” The phase space consists of the different intervals over which the delays are assigned. We can carry out what is known as “autorecurrence analysis” on this single time series, as shown in the bottom-right recurrence plot in Figure 6. From the plot, measures are based on the number of contiguous points, aligned along the diagonals or along the vertical lines. These lines reflect how the system is revisiting regions of its reconstructed phase space, and points are drawn on the plot when the system is within a certain threshold (illustrated by the circle in Figure 6). “Cross” recurrence uses precisely this process of delaying and embedding, but it is done with two time series. In other words, we reconstruct the phase space for two time series separately, then see where each respective series’ trajectories are nearing each other.

This more complex process is most meaningful in the *continuous* case. A visualization of this is shown in Figure 6. As seen here, a continuous signal is being projected into a higher-dimensional space by taking delayed copies of itself. This can also be done with two time series, and observing where these co-visit each other. Typically researchers set a threshold for determining whether the proximity between the time series is “recurrence” (visualized as a sphere in Figure 6). Proximity is calculated as distance between points, and two points are considered as recurring if they fall within a certain *radius*. When dealing with continuous information, in fact, recurrence cannot be calculated just by looking at the match/mismatch between states for every lag, as distances between points results in continuous values. Thus, the additional step involves the evaluation of a radius, which is a threshold constant used to define whether the distance between points is sufficiently small to consider the two points as recurrent. Setting up an optimal radius is not an easy task, as it strongly depends on the type of dataset analyzed, and helpful best practices can be found in Webber and Zbilut (2005).

In the previous section, we calculated cross-recurrence for the simulated dichotomous event series in quite a simple way. The embedding dimension was set to 1, which essentially projects the event series into the same (one) dimension. In addition, we set a threshold to 0, meaning that an event had to match. Though we extracted *RR* measures across the diagonals, here we describe that many measures can be computed from these plots. These measures are derived from the patterns on the plot, often in the form of the diagonal lines reflecting sequences of revisited trajectory regions.

As shown in Figure 4, any individual CRP reveals an array of curious characteristics or “textures” (Eckmann et al., 1987) which can be quantified in various ways (Zbilut and Webber Jr, 1992). In particular, researchers utilize the diagonal line structures to define further measures, because they indicate a sequence of revisitations. The measures that are implemented in our crqa package are as follows:^5^

- recurrence rate (***RR***), the density of recurrence points in a recurrence plot
- percentage determinism (***DET***), the percentage of recurrence points forming diagonal lines in the recurrence plot given a minimal length threshold
- the length of the longest diagonal (***L***_***max***_)
- the average of the diagonal length (***L***)
- the entropy of the diagonal line length distribution (***ENTR***)

From the vertical lines, two more measures can be derived:

- laminarity (***LAM***) is the percentage of recurrence points which form vertical lines given a minimal length threshold
- trapping-time (***TT***) is the mean length of vertical lines

As noted, CRQA can be computed on categorical as well as on continuous-valued time series. In the categorical case, such as a sequence of words, a point recurs when the two series share the same state (i.e., the same word) at two points in time. Recurrence, in this case, can be obtained by means of contingency tables, making cross-recurrence analysis equivalent to lag sequential analysis (Dale et al., 2011b; see also Bakeman, 1997; Bakeman and Quera, 2011 for foundational discussions on the topic). At each lag τ, a contingency matrix *CT* is constructed, where each element of the matrix represents the number of times the pair of objects (*i*, *j*) co-occurs between the two series of events *x* and *y*. More formally: $CT_{i,j}(\tau )=\sum_{t=1}^{t=T-\tau }q(t)$, where *T* is the length of the event series and *q*(*t*) = 1 if *x*(*t*) = *i* and *y*(*t* + τ) = *j*, and *q*(*t*) = 0 otherwise. So, if interlocutor C is uttering the word *cat*, and interlocutor S is instead uttering the word *dog*, we fill the *CT* at the corresponding *i*, *j* position. From *CT*, recurrence *RR* is computed along the diagonal of *CT* by adding the frequencies of looks to the same objects. Obviously a *CT* has the advantage of measuring co-occurrences between all objects at every lag, making it possible to track how different word co-occurrence contributes to recurrence.

**Figure 7:**
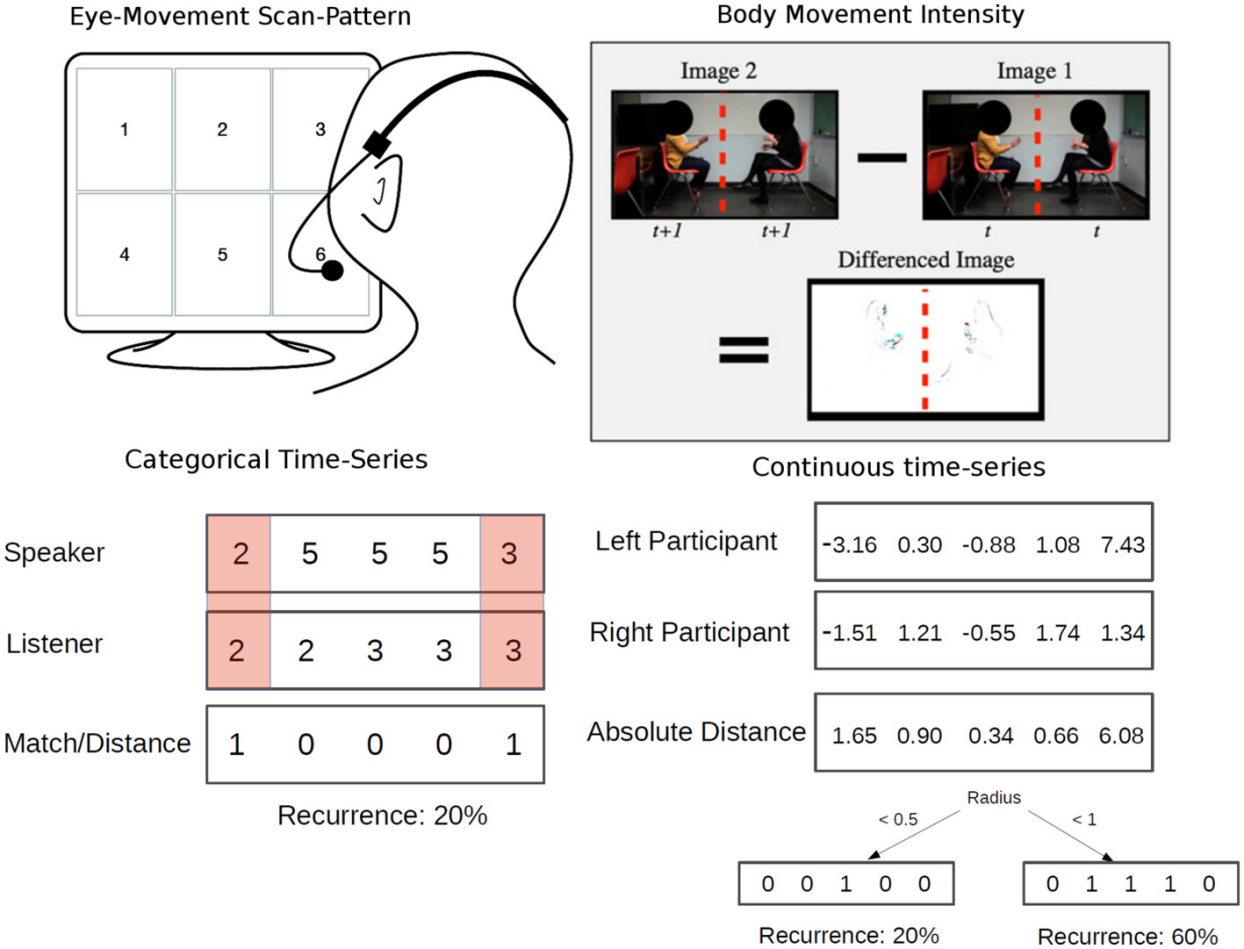
**Data available in crqa**. Eye-movement responses of dyads (speakers and listeners) engaged in dialog from Richardson and Dale (2005) (**left panel**, RDts1, RDts2). Body-movement intensity of the interlocutors engaged in a conversation from Paxton and Dale (2013) (**right panel**, leftmov, rightmov). In the **bottom row** of the figure, we illustrate the concept of recurrence in categorical and continuous time series, and the role played by the radius parameter when the series are not lagged.

Our crqa package implements methods to visualize cross-recurrence patterns on a CRP’s diagonal, extract measures from the whole recurrence plot, as well as compute recurrence on categorical time series by means of a contingency table. In what follows, we describe the functions available in crqa and show their application to example trials taken from published datasets of eye-movement scan-patterns (i.e., a categorical series of fixated objects, Richardson and Dale, 2005) and body movement (i.e., a continuous series representing the overall intensity of body movement of two conversant, Paxton and Dale, 2013). In Figure 7, we show the two example trials data available in crqa (data(crqa)), in simplified form, and provide a visualization of how changing the radius influences the recurrence rate observed when dealing with continuous time series data.

## 4. Functions

In Table 2, we summarize the most important functions available in crqa, their objective, and the output returned. In the Supplementary Material for this paper, we provide the reader with detailed descriptions of each of the functions, their input arguments, output values and a practical R script (testcrqa.R) to replicate all plots and analyses reported below. Exhaustive explanations of each function can also be obtained by using the help() command

**Table 2:** **List in alphabetic order of the most important functions implemented in the crqa package together with a synthetic explanation of their objectives, and the measures outputted**.

**Code**	**Objective**	**Output**
CTcrqa	Recurrence calculated by means of contingency tables on categorical series	Diagonal-wise cross-recurrence profile of the two time series with length equal to the number of delays considered, the maximal recurrence observed, and the delay at which it occurred
calcphi	Recurrence between two categorical time series on a specific state k (Phi-coefficient)	The phi-coefficient profile for state k for all delays considered
crqa	Core cross recurrence function, which examines recurrent structures between time series, which are time-delayed and embedded in higher dimensional space	Several measures (e.g., recurrence rate) computed along the diagonal and vertical lines of the recurrence plot
drpdfromts	Diagonal-wise cross-recurrence of two time series	A diagonal cross-recurrence profile of the two time series with length equal to the number of delays considered, the maximal recurrence observed, and the delay at which it occurred
optimizeParam	Optimal parameters value for CRQA on continuous time series data	Suggested values for radius, number of embedding dimensions and delays
runcrqa	Convenience function wrapping all different methods implemented to compute CRQ	Returns the measures for the method requested
windowdrp	Diagonal-wise cross-recurrence in overlapping windows of a specified size	Windowed cross-recurrence diagonal profile of the two time series, the maximal recurrence observed, and the time-point at which it occurred
wincrqa	Build a cross-recurrence plot in overlapping windows of a specified size	For each window, it returns measures computed along the diagonal and vertical lines of the recurrence plot

Overall, the library provides the user with two main methods of computing cross-recurrence between two time series. First, it includes a faster and simpler calculation of only the diagonal-wise recurrence profile, as demonstrated in the section motivating recurrence above, which contains information both about relative co-visitation and coupling.

The library also includes a second, more detailed method, where a cross-recurrence plot is built for all possible lags, across all states, and several measures of cross-recurrence, e.g., percentage determinism, are extracted. Put simply, this second approach extracts all common CRQA measures.

To compute only the diagonal-wise recurrence profile of the two series, we implemented two functions: drpdfromts and windowdrp. The function drpdfromts extracts the diagonal-wise recurrence profile of two time series. It returns the recurrence observed for different delays, the maximal recurrence observed, and the delay at which it occurred (as demonstrated in the section above).

**Figure 8:**
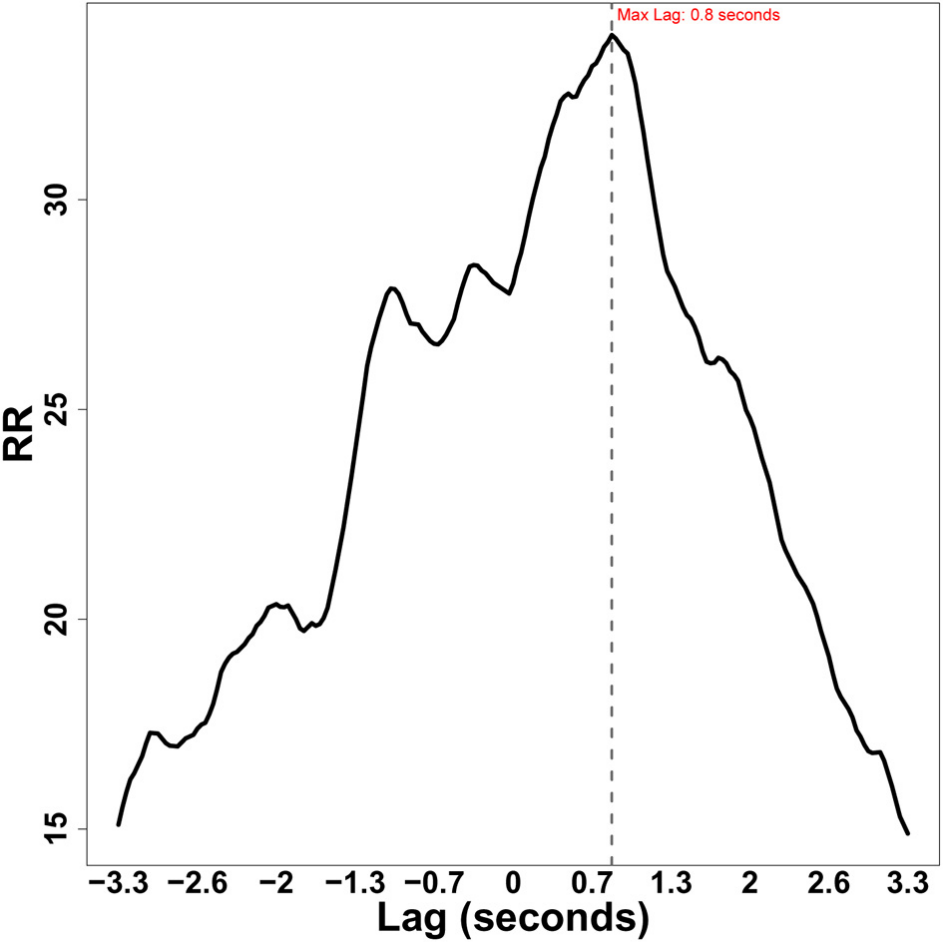
**Diagonal-wise recurrence profile for two eye-movement series (RDts1, RDts2) taken from Richardson and Dale (2005)**.

In Figure 8, we show the diagonal-wise recurrence profile for the two series RDts1,RDts2. Each time series is 2000 datapoints (33 ms each) and are from one pair of a speaker and a listener, respectively, of the dialog dataset by Richardson and Dale (2005). The recurrence profile has the typical leader-follower pattern, where the follower needs a lag of a couple of seconds to be maximally aligned with the speaker’s eye movements. Note, all plots are done using functions external to the crqa package. We refer the reader to the function testcrqa.R available as Supplementary Material of this paper.

When using drpdfromts, for categorical sequences, the radius should be set to a very small value (near 0, e.g., 0.001). As the categories in the sequence (e.g., one of the six possible character being looked at) are recoded into numbers (e.g., 1), setting the radius to very small value would make only the distance between the same category, i.e., 0, be accepted. By changing the datatype argument to “continuous,” the function would compute cross-recurrence between time series of continuous data, so the series will be maintained as numerics. Also for continuous data, we would need a value for the argument radius. However, the value of the radius would have to be tailored to the data observed, because each dataset has its own idiosyncratic properties, e.g., body movement vs. eye movements. Below, we discuss this issue further, namely choosing starting parameter values for continuous data. We show an early alpha version of a function that can perform an optimization routine to estimate these parameters, based on phase-space reconstruction principles (Marwan et al., 2007) (see function optimizeParam).

The function windowdrp, instead, has similarity to windowed cross-correlation analysis as in Boker et al. (2002), and tracks how cross-recurrence values evolve over the time course. In particular, CRQA measures are calculated in overlapping windows of a specified size for a number of delays smaller than the size of the window. In every window, the recurrence value for the different delays is calculated. A mean is then taken across the delays to obtain a recurrence value in that particular window. Tracking recurrence over the time course helps us establishing how the agreement between the two interlocutors develops, as the interaction progresses. We reuse the eye-movement categorical responses RDts1, RDts2, to display how windowed cross-recurrence between a speaker and a listener evolves as a function of time.

**Figure 9:**
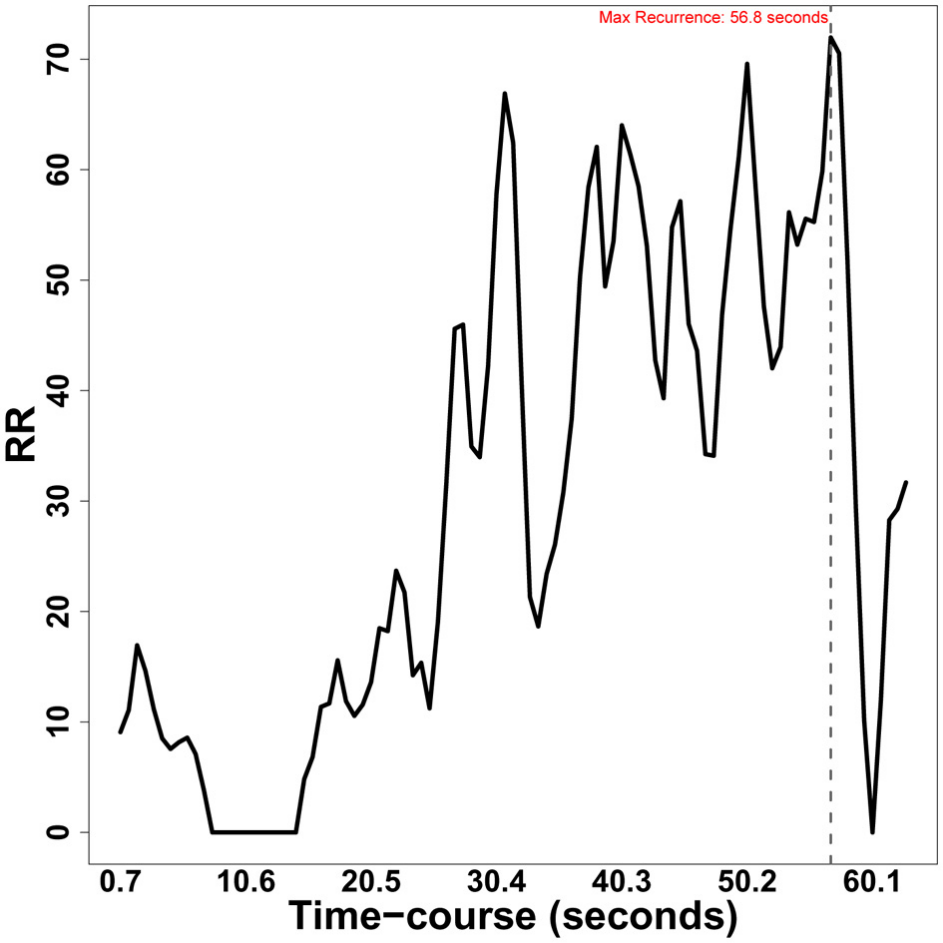
**Window cross-recurrence of the two eye-movement series (RDts1, RDts2) from Richardson and Dale (2005)**.

In Figure 9, we can see that about half the time course, the amount of overall recurrence increases, and then fluctuates around the same value till almost the end where it drops. The dyads became more coupled, then recurrence quickly drops as the speaker concludes. Also windowdrp can be applied to continuous data by setting up the appropriate datatype and radius argument, as just described.

More detailed measures characterizing the cross-recurrence of the two time series can be obtained by using crqa. crqa is the core function of the package, and examines recurrent structures between time series, which are time-delayed and embedded in higher dimensional space. The approach compares the phase space trajectories of two time series in the same phase-space when delays are introduced. A Euclidean distance matrix between the two series, delayed and embedded is calculated^6^. On the distance matrix, a recurrence plot is derived by taking all points below a certain radius threshold as recurrent (refer to Figure 7 for a simplified illustration of the radius). The function implements a Theiler window parameter (tw), which is used to specify the diagonal lines of the recurrence plot to be ignored, with 1 indexing the main diagonal. This parameter is particularly useful when auto-recurrence is computed, as there can be autocorrelation structure of the time series with itself around the main diagonal (e.g., slow-moving continuous time series). However, the tw parameter should be set to 0 in CRQ, as two time series are different and they are especially synced along the main diagonal (i.e., the LOC). Several measures representative of the interaction, e.g., recurrence rate (RR), are extracted from the recurrence plot (as explained in Principles, above).

**Figure 10:**
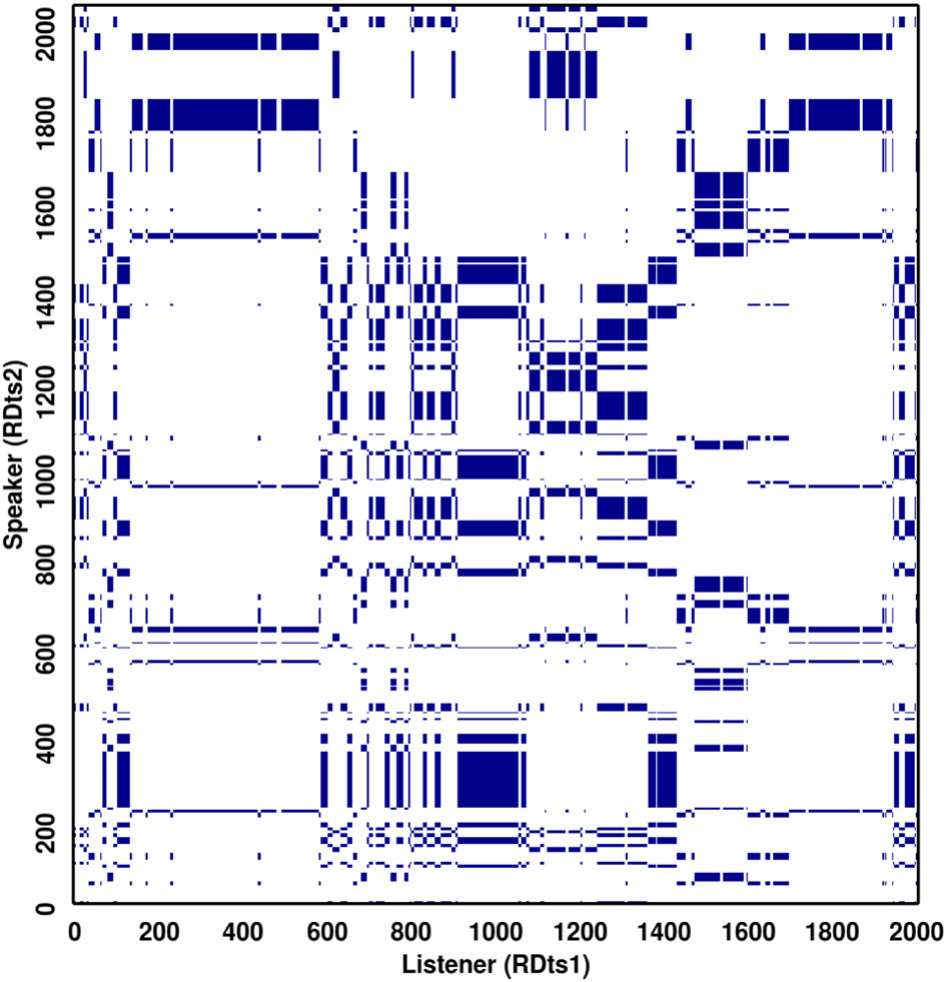
**Recurrence Plot of the two eye-movement series (RDts1, RDts2) from Richardson and Dale (2005)**. The recurrent points are marked with blue color, whereas the non-recurrent points are left blank. The values obtained on the measures for this plot are: *REC* = 12.52; *DET* = 98.95; *Lmax* = 124; *L* = 11.3; *ENTR* = 3.2; *LAM* = 99.7; *TT* = 20.6. Usually, these values are interpreted relatively, by comparing one condition to another condition in an experiment. In general, *DET* will be higher than *REC*, with *DET* often quite high (90% or higher) and *REC* considerably lower (10% or less), so 12% would be considered relatively high.

In Figure 10, we show the cross-recurrence plot obtained using the two-time series (RDts1, RDts2) from Richardson and Dale (2005). On the diagonal lines, we observe the pattern of interaction between the two series. The measures characterizing it are *RR*, percentage determinism (*DET*), average and maximal diagonal length (*L* and *L*_*max*_), and entropy are calculated. On the vertical lines, we observe the stability of the two series, and relative independence of recurrence over a particular state. The measures characterizing this information are laminarity and trapping-time (*LAM* and *TT*).

A challenging aspect of computing CRQA is finding appropriate values for the three parameters radius, delay, embed, especially when dealing with continuous time series. The function optimizeParam implements an iterative procedure that in three steps attempts to find such values. In particular, the function first identifies a delay that accommodates both time series by finding the local minimum where mutual information between them drops, and starts to level off (Shockley, 2005; Marwan et al., 2007). When one time series has a considerably longer delay than the other, the function selects the longer delay of the two to ensure that new information is gained for both. When the delays are close to each other, the function computes the mean of the two delays. Then, as a second step, the function determines the optimal number of embedding dimensions by using false nearest neighbors, and checking when it bottoms out (i.e., there is no gain in adding more dimensions). If the embedding dimensions for the two time series are different, the algorithm selects the higher embedding dimension of the two to make sure that both time series are sufficiently unfolded. Finally, it determines the radius to use for recurrence by selecting the first radius that yields 2–5% RR. In particular, in order to explore an exhaustive range of values while keeping the computation tractable, the algorithm generates a sample of equally spaced possible radius values, returning from ≈25–0% RR. The radius is iteratively explored till RR between 2–5% is found. The algorithm includes parameters to modify the granularity of the radius being generated, as well as, the size of the sample. Applied on the continuous body-movement intensity *z*-score of two conversant (leftmov, rightmov) taken from the dataset of Paxton and Dale (2013), we obtain: *radius* = 5.74, *embedding dimension* = 4, *delay* = 12^7^. Obviously, this procedure should be iterated over a consistent sample of the data, such that a more precise estimate for the values of the parameters can be obtained.

The crqa package also provides the user with a wrapper, runcrqa, which calls all the methods implemented, such as the simple profile recurrence (drpdfromts) or the more extensive analysis of the cross-recurrence plot (crqa) both when delays are introduced (method = ’profile’) and for a time-course analysis of recurrence (method = ’window’). The different methods are called using a list par of arguments, according to the type of analysis to be performed (refer to the Supplementary Material, R code 5, for more details about the arguments and output).

The last function described in this paper is CTcrqa, which is used to compute cross-recurrence on categorical sequences by means of contingency tables (Bakeman, 1997; Dale et al., 2011b). First, it finds the common states, or categories, shared by the two time series, then it builds up a contingency table (CT) counting the co-occurrences of different sets of states between the two series. For example, in Richardson and Dale (2005) six possible characters could be fixated on the visual array during the task. These are nominally coded 1–6. This contingency-table approach builds a 6 × 6 table, the cells of which count the number of times speaker/listener were looking at the characters corresponding to that row/column for a given portion of the time series (or, alternatively, the entire time series). The diagonal of the CT is where the recurrence profile is calculated, as along the diagonal, the states are identical.The advantage of this method is to be able to track co-occurrences of all states involved for each delay introduced. Such values could be potentially used to estimate probability distribution of co-occurrences between states of the two series analyzed, drawing bridges to other sophisticated analytic frameworks, such as lag-sequential analysis (Bakeman, 1997).

When computing recurrence between categorical sequences, we might be specifically interested in a certain object or state. In an eye-tracking dialog experiment, for example, we might be interested in how looks to a specific target object recur between speakers and listeners. Likewise, in the speech produced by the dyads as they interact, we might be interested in the usage of a specific word referring to that object. The function calcphi precisely calculates how recurrence on a specific object between two-series changes when the series are delayed. In particular, the phi(k) coefficient increases with the frequency of matching recurrence on the same state (k; k) and away from this state (not k; not k) between the two time series. On the other hand, phi(k) decreases with the frequency of mismatching objects (k; not k, and vice versa).

**Figure 11:**
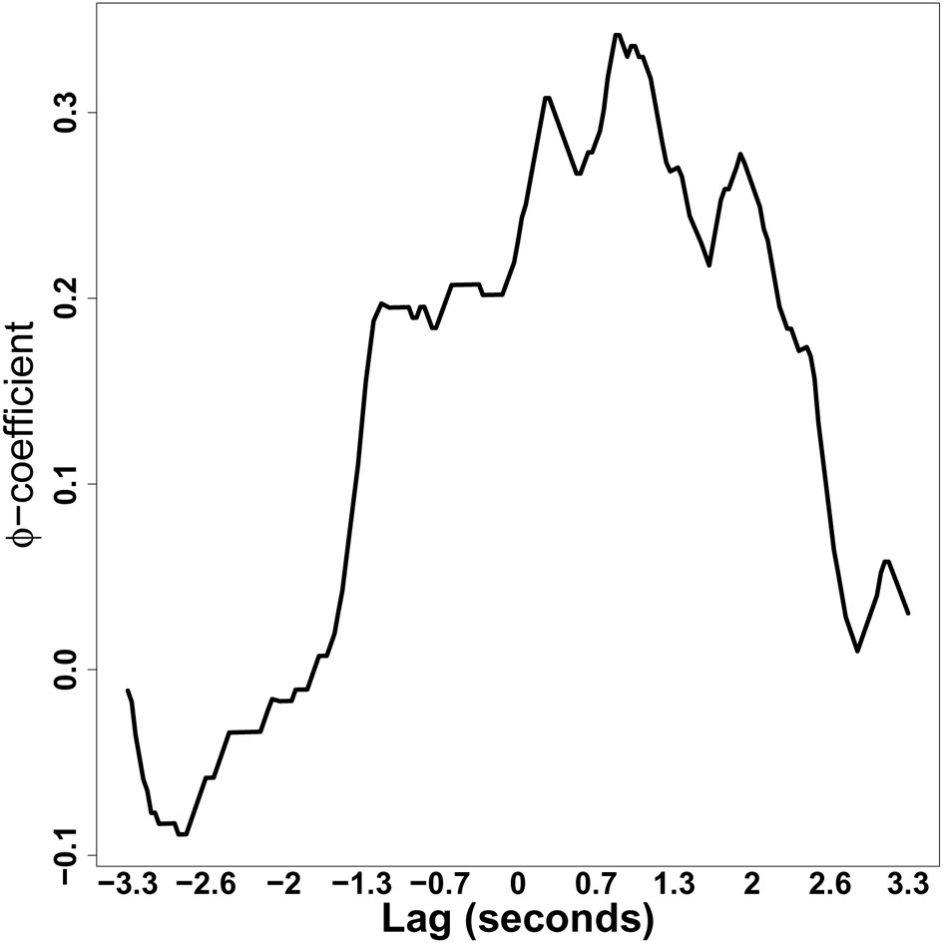
**Phi-coefficient plot of a particular object for the two eye-movement series (RDts1, RDts2) from Richardson and Dale (2005)**.

In Figure 11, we show the phi-coefficient for a particular object, coded as 5, looked at in the two series (RDts1, RDts2) from Richardson and Dale (2005). This object was one of six quadrants depicting TV-series characters, that participants had to discuss (refer to Figure 7 for a visualization of the type of data). In line with Figure 8, we observe the characteristic speaker-leading pattern, whereby the listener takes about one-second to look at object 5, after the speaker has mentioned it.

## 5. Test of efficiency and consistency

We ran 20 iterations and generated two dichotomous time series with parameters *P*(*C*) = 0.08, *P*(*S*) = 0.05, *P*(*C*|*C*) = *P*(*S*|*S*) = 0.05, and *P*(*S*|*C*) = 33 (refer to Table 1 for details) of increasing size (from 250 to 3000, steps of 250; 11 different unique size). In a total of 220 simulations, we measure the elapsed user time taken to build a CRP and extract from it the following seven measures: *RR* (recurrence rate), *DET* (percentage determinism), *L*_*max*_ (length of longest diagonal line), *L* (average diagonal length), *ENTR* (entropy of diagonal lengths above line cutoff, *min* > 2), *LAM* (laminarity of vertical lines) and *TT* (trapping time). For each of the measures, normalized to range between 0 and 1, we compute mean and standard deviation for the absolute distance between the values obtained by R and MATLAB code. Moreover, in order to assess whether the measures obtained with R and MATLAB account for the same variance in the data, we test for correlation and report the *p*-values observed. Obviously, both packages are tested on the same dataset of simulated time series. Simulations using R (3.0.2, “Frisbee Sailing”) and MATLAB (2012) were run with a standard PC laptop machine equipped with an Intel dual core (32 bit), 2.20 GHz, 2.8 GiB RAM, on a Linux OS (Ubuntu 12.04). When calling crqa from the crqtoolbox (version 5.15) in MATLAB by Marwan (2013), we suppressed GUI and other outputs from being printed (i.e., “silent,” “nogui”)^8^.

**Figure 12:**
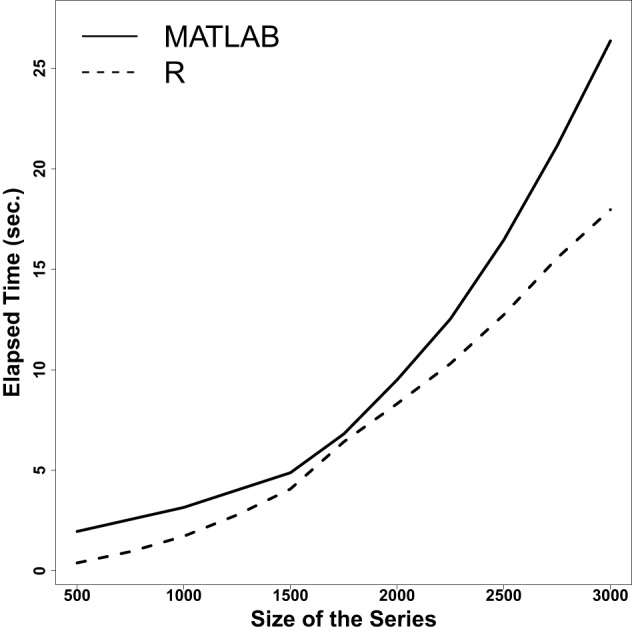
**Elapsed user time to extract CRQ measures on simulated dichotomous time series of increasing lengths using crqa in R and crqtoolbox in MATLAB**. Means over 20 iterations are shown as lines. The programming language of the library is identified using line type.

In Figure 12, we plot mean elapsed user time (*y*-axis) as a function of sequence lengths. As expected, both libraries demand more time to finish the computation as the time series get longer. However, the R implementation outperforms the MATLAB version for increasing size. Crucially, when comparing their performance, we obtain a mean absolute difference of 0.0002 across all measures over 220 simulations. Moreover, all measures correlated at ρ = 1 with a significance of *p* < 0.00001.

These results show that the performance and results obtained with the crqa library in R are 100% comparable to the benchmark MATLAB crptoolbox toolbox by Marwan (2013). Obviously, the consolidated MATLAB toolbox provides the user with an extremely handy GUI, as well as numerous other functionalities to visualize the results and compute alternative measures from the recurrence plots. In this respect, the MATLAB toolbox by Marwan, et al., can still be considered the benchmark for recurrence analyses. However, we believe that our library can be expanded in the future to integrate more functionalities; and as R is a free software for statistical computing, such effort would be certainly sustained by its community of committed users.

## 6. General discussion

Humans are complex systems, dynamically and interactively exchanging information with their surrounding environment. The most prominent manifestation of such dynamism is observed when humans talk with each other, where the behavior of a single individual engaged in the interaction adapts and aligns with the behaviors of the other individuals that are taking part to the interaction (e.g., Pickering and Garrod, 2004).

The alignment occurring between two interacting individuals has been classically quantified using an aggregative approach, i.e., by correlating frequencies of occurrences of a certain behavior (Bargh and Chartrand, 1999). In language science, the aggregative approach has been the most prominent, where alignment has been measured as the number of common linguistic structures (e.g., lexical, syntactic) used by two interlocutors engaged in a communicative task (Brennan and Clark, 1996; Haywood et al., 2005; Branigan, 2007).

However, alignment has an intrinsic temporal structure, as it unfolds over a sender-receiver feedback mechanism, e.g., turn-taking in dialog. Such temporal dependence of alignment has been clearly observed taking place on several “macro” behaviors, such as postural sways (e.g., Shockley et al., 2003; Louwerse et al., 2012), “micro” behavior, such as eye-movement (e.g., Richardson and Dale, 2005), as well as linguistic analyses such as words or letters (e.g., see Orsucci et al., 2006).

The statistical modeling approach used to capture how a dynamical system interactively evolves over time is recurrence analysis (Zbilut et al., 1998; Marwan and Kurths, 2002). This approach aims at quantifying the temporal organization of interacting signals by uncovering the *phases* where such signals are recurring, i.e., they are on the same state; and the *delays* over which recurrence develops.

In this paper, we first empirically motivated the crucial difference between correlation (typically used in the aggregative approach), and co-visitation (typically used in the recurrence approach), and demonstrated that the latter offers a distinct analytic framework. Cross-recurrence quantification analysis is an approach to investigate alignment on a large range of behavioral phenomena, quantifying a range of dynamic relationships that hold between two time series. In particular, we generated binary dichotomous time series, where the probability of certain event to occur in one time series is conditioned to the probability that the event will occur in the other time series. In practice, we simulated an extremely simple interactive system, which can resemble statistical characteristics of real behaviors, such as nodding, or smiling. By using cross-recurrence quantification analysis, we demonstrated that we can capture the same patterns as an aggregative approach, and go beyond that by uncovering the temporal phases during which the interaction takes place.

The advantages of cross-recurrence analysis over more classic approaches to the study of dynamical systems have called the attention of many researchers across different fields in cognitive science. Such attention is, in fact, reflected by the amount of recently published work, spanning several topics, where cross-recurrence quantification analysis is used (e.g., Fusaroli et al., in press).

The most frequently used software to perform this type of analysis is the MATLAB toolbox crptoolbox by Marwan (2013). Even though crptoolbox is an excellent tool to perform cross-recurrence analysis, the research community still lacks an efficient open-source package for the R platform. In the second part of this paper, we explained more formally the principles of CRQA analysis, and described the R package crqa, which provides to a broad audience several basic, and more advanced, tools to carry out cross-recurrence quantification analysis.

Our package contains functions to quantify cross-recurrence at different levels of analyses. In particular, drpdfromts constructs diagonal-wise recurrence profiles of the two time series across different lags, while windowdrp returns a windowed cross-recurrence analysis where recurrence is tracked over the time-course. These two functions just look at the overall cross-recurrence shape. crqa instead performs a complete analysis of the cross-recurrence plot returning several measures, such as recurrence rate, percentage determinism, etc. characterizing the dynamics of interaction taking place in the system. By using principles of phase-space reconstruction (Marwan et al., 2007), our library also includes an alpha function, optimizeParam, to estimate “optimal” values for the parameters of *radius*, *delay*, and number of *embedding* dimension. Moreover, the library makes available a function to compute cross recurrence analysis on categorical data by means of contingency tables CTcrqa. The advantage of this function, yet to be fully exploited, is that it potentially returns a co-occurrence matrix of all states of the two series at each delay. Such co-occurrence statistics might be integrated in future development of the crqa to better estimate recurrence properties of categorical series.

After presenting the most important functions included in our package, we compared its computational efficiency and consistency with the benchmark MATLAB toolbox (crptoolbox) developed by Marwan (2013). By using simulated dichotomous time series, we demonstrated that our library can be computationally more efficient than its MATLAB rival. In particular, we observed that our R library maintained a better elapsed user time as a function of increasing set sizes. Besides being computationally efficient, our package returns measures, which are completely consistent with those generated by crptoolbox.

Even though our crqa package achieves remarkable performance, it cannot yet substitute the older and proven crptoolbox by Marwan (2013). In fact, crptoolbox implements a very handy GUI, integrates many functionalities for plotting, and it includes additional recurrence measures. Thus, our package will complement rather than substitute crptoolbox, by providing the open-source alternative for computing cross-recurrence to the wide community of researchers using R as their statistical programming language. Moreover, we believe that the functionalities available in the package will expand in the future with the contribution of its community of users.

### Conflict of interest statement

The authors declare that the research was conducted in the absence of any commercial or financial relationships that could be construed as a potential conflict of interest.

## Footnotes

^1^Readers can also consult Norbert Marwan’s http://recurrence-plot.tk/programmes.php for additional resources and software tools.

^2^Note that the side on which this lag occurs is chosen by the experimenter, and simply reflects the fact that *one* system is leading the other in some way.

^3^It is important to note here that in practice, the 0 event codes are recoded differently for two time series, as distinct “non-event” codes, such as 11 or 12 (for example) to make sure that these non-events do not produce recurrence points on the plot.

^4^Below we also discuss contingency table analysis, and the importance of lag sequential analysis as in Bakeman and Quera (2011); this provides more information about inferential contexts, such as the log-linear functions. It historically precedes and is a very strong basis for doing event-based lag analysis, and, at present, it still offers a more developed statistical basis for making inferences in the categorical case.

^5^Formal definitions of these measures can be found in Marwan et al. (2007).

^6^The current version of the package only implements the Euclidean distance, but other metrics can be used.

^7^As this procedure involves sampling the result for the radius might slightly vary for different runs.

^8^Note, even silencing all outputs, a waiting box was automatically launched, and could not be suppressed.
